# RNA-seq of Isolated Chromaffin Cells Highlights the Role of Sex-Linked and Imprinted Genes in Adrenal Medulla Development

**DOI:** 10.1038/s41598-019-40501-0

**Published:** 2019-03-08

**Authors:** Wing Hei Chan, Masayuki Komada, Toshiaki Fukushima, E. Michelle Southard-Smith, Colin R. Anderson, Matthew J. Wakefield

**Affiliations:** 1 Department of Anatomy and Neuroscience, University of Melbourne, Melbourne, Australia; 2 Cell Biology Center, Institute of Innovative Research, Tokyo Institute of Technology, Tokyo, Japan; 3 Department of Medicine, Vanderbilt University School of Medicine, Nashville, Tennessee USA; 4 Melbourne Bioinformatics, University of Melbourne, Melbourne, Australia; 5 Walter and Eliza Hall Institute, Parkville, Australia

## Abstract

Adrenal chromaffin cells and sympathetic neurons synthesize and release catecholamines, and both cell types are derived from neural crest precursors. However, they have different developmental histories, with sympathetic neurons derived directly from neural crest precursors while adrenal chromaffin cells arise from neural crest-derived cells that express Schwann cell markers. We have sought to identify the genes, including imprinted genes, which regulate the development of the two cell types in mice. We developed a method of separating the two cell types as early as E12.5, using differences in expression of enhanced yellow fluorescent protein driven from the tyrosine hydroxylase gene, and then used RNA sequencing to confirm the characteristic molecular signatures of the two cell types. We identified genes differentially expressed by adrenal chromaffin cells and sympathetic neurons. Deletion of a gene highly expressed by adrenal chromaffin cells, NIK-related kinase, a gene on the X-chromosome, results in reduced expression of adrenaline-synthesizing enzyme, phenyl-N-methyl transferase, by adrenal chromaffin cells and changes in cell cycle dynamics. Finally, many imprinted genes are up-regulated in chromaffin cells and may play key roles in their development.

## Introduction

Neural crest cells give rise to adrenal chromaffin cells and sympathetic neurons^1–3^, which show many molecular similarities including their ability to synthesize and release catecholamines. A recent study^4^ has shown that sympathetic neuroblasts and developing chromaffin cells do not share an immediate common precursor. Instead, chromaffin cells arise from neural crest-derived precursors that accompany the preganglionic nerves, while sympathetic neuroblasts arise from a separate population of neural crest cells.

Despite their separate origins, both chromaffin cells and sympathetic neurons can give rise to neuroblastoma, the most common solid tumor in infants and both cell types share a catecholaminergic phenotype^5^. We sought to understand the molecular mechanisms that underlie the separate developmental histories and also the many similarities between the two cell types. While a significant amount is known about the transcriptional networks that underlie sympathetic neuron development^6^, little is known about equivalent mechanisms in adrenal chromaffin cells.

One gene previously noted to be upregulated in developing adrenal chromaffin cells is Delta-like 1 homolog (*Dlk1)*^7–10^, an imprinted gene. Genomic imprinting is an epigenetic control process that specifically silences one of the parentally-inherited alleles in a gene, as a result causing gene expression in a parental origin-specific manner^11^. It is known that imprinted genes can be regulated in a developmental stage- and tissue-specific manners^12,13^. The imprinted genes, *Zdbf2*^14^, *Th*^15^ and *Cdkn1c*^16^, were found by Furlan *et al*.^4^ to be upregulated in developing adrenal chromaffin cells. Thus, a further goal of this study is to ascertain whether imprinted genes are generally upregulated in developing adrenal chromaffin cells.

We examined developing adrenal chromaffin cells and sympathetic neuroblasts from E12.5 in mice. Separation of embryonic adrenal chromaffin cells from sympathetic neuroblasts is technically challenging due to their phenotypic similarity and low numbers. In our previous study^17^, TH and CART were revealed as potential makers for the early separation of sympathetic neuroblasts and adrenal chromaffin cells in E12.5 mice because of their differential expression levels; sympathetic neuroblasts cells are relatively low in TH-immunoreactivity while the adrenal chromaffin cells have significantly higher TH-immunoreactivity, reflecting differences in *Th* RNA expression^4^. In addition, only sympathetic neuroblasts are immunoreactive for the neuropeptide, CART (Cocaine and Amphetamine Regulated Transcript) from E12.5 to E13.5. Therefore, in the present study we used TH-Cre activation of enhanced yellow fluorescent protein (EYFP) expression in transgenic mice coupled with fluorescence-activated cell sorting (FACS) to isolate and collect sufficient number of sympathetic neuroblasts and adrenal chromaffin cells at E12.5 for transcriptomic analysis by RNA sequencing. This allowed the assessment of all differentially expressed genes, and the identification of potentially important transcription and cell signaling genes. Subsequent studies tested the leading candidate gene for a role in chromaffin cell development along with assessing the expression of imprinted genes.

## Results

### Differential EYFP Expression in Sympathetic Neuroblasts and Adrenal Chromaffin Cells

**Figure 1:**
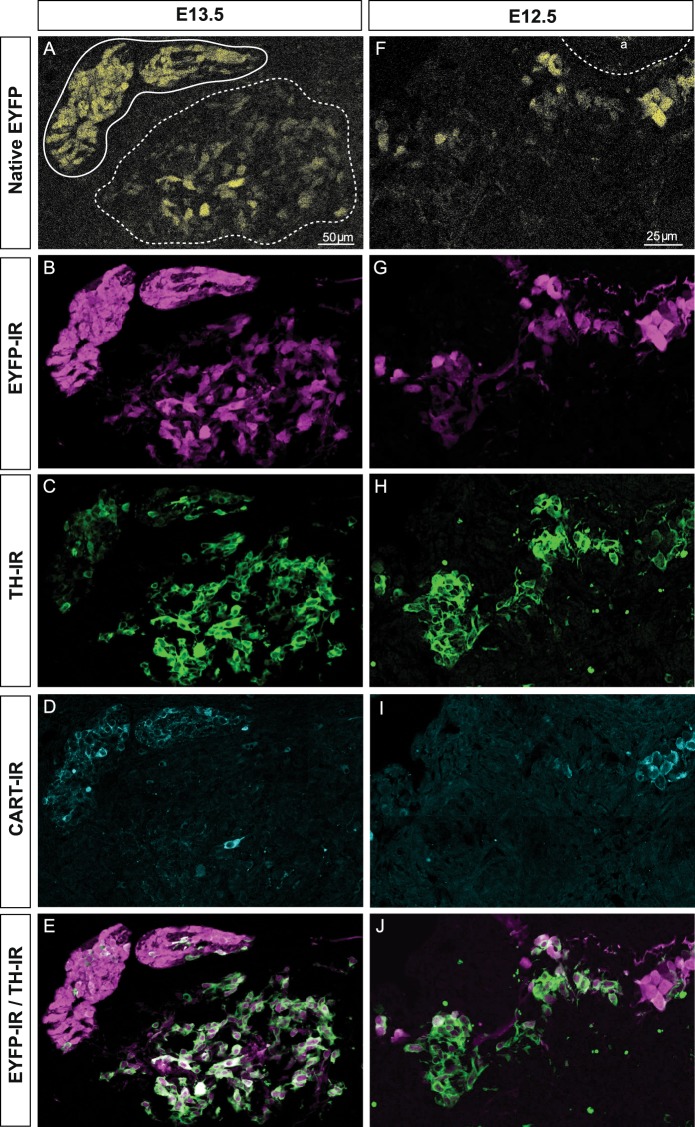
Immunostaining of transverse sections through the adrenal region of TH-Cre::R26R-EYFP mouse embryos at E13.5 (**A**–**E**) and E12.5 (**F**–**J**). A shows the native EYFP (yellow) signal after fixation of TH-Cre::R26R-EYFP mouse embryos at E13.5, the prevertebral suprarenal ganglion (solid line) and the adrenal medulla (dashed line) marked. EYFP-immunoreactivity for the same section is shown in (**B**), TH-immunoreactivity in (**C**) and CART-immunoreactivity in (**D**). (**E**) Is a merge of images (**B**,**C**). Note that TH immunoreactivity shows the reverse pattern of intensity to both native EYFP and EYFP-immunoreactivity. (**F**–**J**) is an equivalent region from an E12.5 embryo as (**A**–**E**). The dorsal aorta (a) is indicated. Note that differential expression of TH-driven EYFP was observed in that some TH-expressing cells were brighter in EYFP than the others, but there was no obvious anatomical segregation of cells differentially expressing EYFP.

We have shown that TH immunoreactivity in developing chromaffin cells is significantly higher than in sympathetic neuroblasts^17^. We sought to separate developing chromaffin cells from sympathetic neuroblasts based on this difference using TH-Cre::R26R-EYFP reporter mice. In E13.5 mice (Fig. 1A–E), where developing chromaffin cells and sympathetic neuroblasts were anatomically distinct, surprisingly the native EYFP signal (and EYFP immunoreactivity seen using a green fluorescent protein antiserum) in the adrenal gland anlagen was weaker than in the suprarenal and other prevertebral ganglia (Fig. 1E), the inverse of the staining intensity difference seen with antisera to TH^17^. In E12.5 TH-Cre::R26R-EYFP mice (Fig. 1F–J), where anatomical boundaries between developing chromaffin cells and sympathetic neuroblasts were much less distinct, there was also heterogeneity in both native EYFP and EYFP immunoreactivity. EYFP+ cells with both high and low levels of expression were usually intermingled without clear anatomical boundaries.

**Figure 2:**
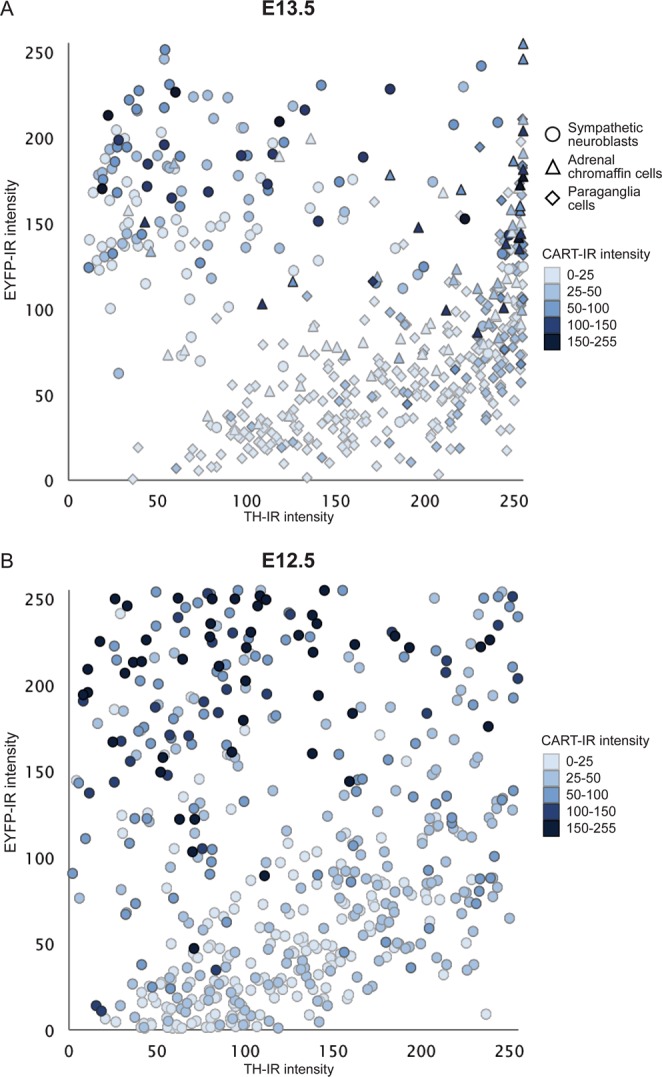
Scatter plot of the relative fluorescent intensity of TH-IR versus that of EYFP-IR in individual sympathetic neuroblasts and adrenal chromaffin cells in sections through the abdomen of E13.5 (**A**) and E12.5 (**B**) TH-Cre::R26R-EYFP mice. CART-IR fluorescence intensity in each cell is shown by the shading of each symbol (darker is more intense CART-IR). Relative fluorescent intensity for TH-IR (X-axis), EYFP-IR (Y-axis) and CART-IR (shading) were measured on a 0–255 scale (0 = no fluorescence, 255 = maximum fluorescence). The upper left quadrant of each plot contained TH-IR _Lo_/EYFP-IR_Hi_ cells while lower right quadrant contained TH-IR_Hi_/EYFP-IR_Lo_ cells. For E13.5, cells are identified and categorized into sympathetic neuroblasts (○), paraganglia (△) and adrenal chromaffin cells (◊) based on anatomical segregation into adrenal medulla or sympathetic ganglia. On E12.5, it was not possible to discriminate cell type by anatomical organisation and the cells are unclassified. However, the pattern observed in the plot is identical to that seen at E13.5, leading to the conclusion that cells that are TH-IR_Lo_/EYFP-IR_Hi_ and which also express CART-IR are likely to be sympathetic neuroblasts while TH-IR_Hi_/EYFP-IR_Lo_ cells lacking CART-IR are likely to be adrenal chromaffin cells. For E13.5, n = 3 embryos, 521 cells and for E12.5, n = 2 embryos, 505 cells.

We then examined whether cells expressing high levels of EYFP from the TH transgene (EYFP+_Hi_) corresponded to sympathetic neuroblasts while cells expressing low levels of EYFP (EYFP+_Lo_) corresponded to developing chromaffin cells. We quantified and plotted the relative fluorescence intensity for TH-IR against EYFP-IR for each cell in the abdominal region of E13.5 and E12.5 TH-Cre::R26R-EYFP mice (Fig. 2). The distributions of both TH and EYFP-IR immunofluorescence at E13.5 (Fig. 2A) appeared largely bimodal, with adrenal chromaffin cells clustering in the lower right region of the graph (TH-IR_Hi_/EYFP-IR_Lo_ cells) and sympathetic neuroblasts from the suprarenal ganglia clustered in the upper left region of the graph (TH-IR_Lo_/EYFP-IR_Hi_ cells). Chan *et al*.^17^ showed that immunoreactivity to CART was specific to sympathetic neuroblasts and not present in developing chromaffin cells. CART-immunoreactivity was present almost exclusively in TH-IR_Lo_/EYFP-IR_Hi_ cells (Fig. 2A), confirming that the levels of EYFP immunofluorescence can be used to separate the two cell types. A handful of cells, previously identified as extra-adrenal chromaffin or paraganglionic cells^17^, expressed CART-IR in addition to high levels of EYFP-IR and TH-IR (Fig. 1A–D).

At E12.5, where the anatomy cannot be reliably used to separate the developing chromaffin cells from sympathetic neuroblasts, there were again two distinct types of EYFP+ cells; one with high intensity of EYFP-IR (EYFP-IR_Hi_) and the other low intensity of EYFP-IR (EYFP-IR_Lo_), intermixed around the dorsal aorta (Fig. 1F–J). When EYFP-IR was plotted against TH-IR for each cell, they segregated into two populations, as seen in E13.5 embryos (Fig. 2B). Again, when CART-IR (a marker of sympathetic neuroblasts) was considered, it was expressed predominantly by cells with a TH-IR_Lo_/EYFP-IR_Hi_ phenotype that lay in the upper left part of the distribution (Fig. 2B). These data show that separation of developing adrenal chromaffin cells and sympathetic neuroblasts is possible in TH-Cre::R26R-EYFP mice as early as E12.5 on the basis of their differential EYFP expression. This was confirmed below, using RNA sequencing analysis after FACS isolation.

### FACS Isolation of Live Sympathetic Neuroblasts and Adrenal Chromaffin Cells

**Figure 3:**
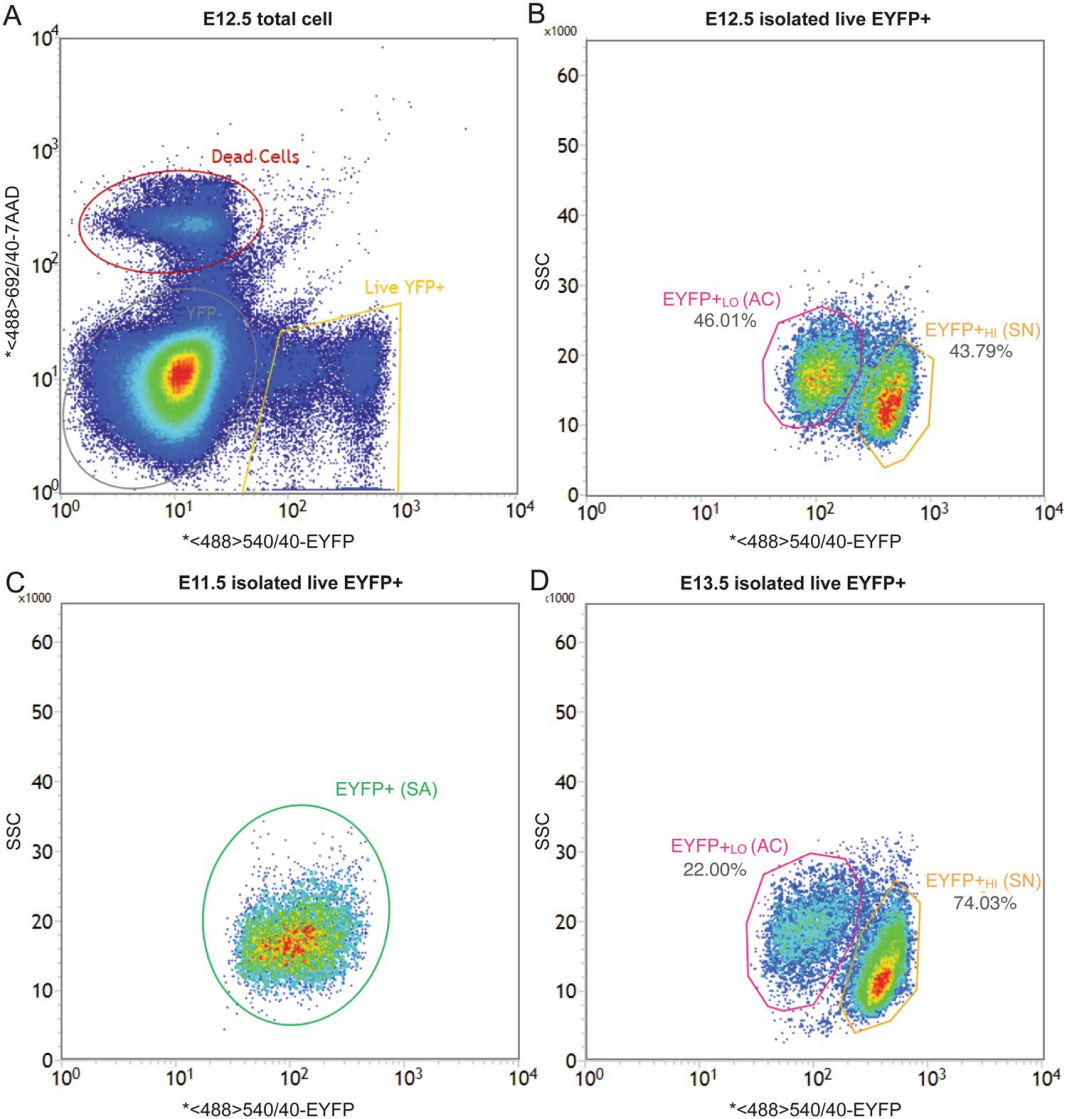
Representative FACS plots of cells in TH-Cre::R26R-EYFP mice embryo at E11.5–E13.5. Cells dissociated after dissection were sorted by FACS for EYFP fluorescent intensity and viability (7-AAD). (**A**) Plot of all cells at E12.5, showing clear separation of living EYFP+ cells (yellow gate) from living EYFP− cells (grey gate) and dead cells (red gate). (**B**) Plot of living E12.5 EYFP+ cells showing the two cell clusters with similar population size separated based on EYFP fluorescent intensity and SSC. EYFP+_Lo_ (pink gate) are presumptive adrenal chromaffin cells (46.01%) while EYFP+_Hi_ (orange gate) are presumptive sympathetic neuroblasts cells (43.79%). (**C**) Plot of living E11.5 EYFP+ cells showing a homogeneous population (green gate). (**D**) Plot of living E13.5 EYFP+ cells showing a similar separation to the E12.5 cells except the population size of sympathetic neuroblasts were about 3 times more than adrenal chromaffin cells.

Cells from E11.5, 12.5, and 13.5 TH-Cre::R26R-EYFP mice were subjected to FACS based on native EYFP fluorescence intensity and 7-AAD staining to exclude dead or damaged cells (Fig. 3A) and then on side-scattered light (SSC, Fig. 3B–D). Consistent with our hypothesis that differences in EYFP intensity can separate developing adrenal chromaffin cells from sympathetic neuroblasts in embryonic TH-Cre::R26R-EYFP mice, E12.5 was the earliest stage at which two different populations of EYFP+ cells could be separated (Fig. 3B,C). Careful gating at E12.5 and E13.5 in the FACS eliminated the small number of cells that lay intermediate between the two populations. Gating also eliminated cells showing very high intensity in both EYFP and SSC, as they likely represent the presumptive paraganglionic cells. At E11.5, EYFP+ cells formed a homogeneous population without obvious segregation into EYFP+_Hi_ and EYFP+_Lo_ populations (Fig. 3C). Our approach enables separation of progenitor cells that give rise to chromaffin cells and sympathetic neurons as early as E12.5 and was used to identify candidate genes involved in the development of the two cell types.

### RNA sequencing analysis

**Figure 4:**
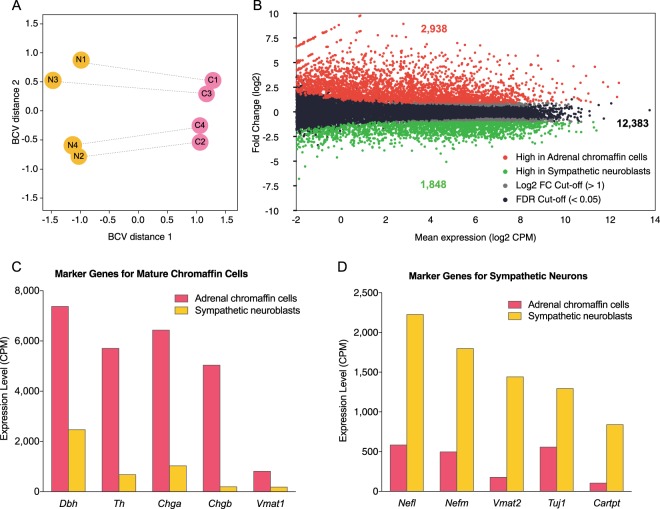
(**A**) Principal component analysis plot of biological coefficient of variation (BCV) showing the transcriptome profiles from each of 4 paired (connected by line) E12.5 samples separated clearly by the biological effect of interest i.e. cell types (Dimension 1, X-axis). The effect size of technical batch effect on replicates (Dimension 2, Y-axis) was small, about half of the biological effect. (**B**) MA-plot of transcriptomic profile in adrenal chromaffin precursor cells versus sympathetic neuroblasts. RNA sequencing analysis of adrenal chromaffin cells and sympathetic neuroblasts revealed 4,786 differential expressed genes with fold change >2 and false discovery rate (FDR) < 0.05 out of 17169 annotated genes, that 2,938 genes expressed higher in adrenal chromaffin cells (red) and 1,848 genes were expressed higher in sympathetic neuroblasts (green). (**C**,**D**) The mRNA expression levels of the ten representative cell type-specific marker genes are shown. Markers for chromaffin cells were all expressed at higher level in the adrenal chromaffin cell transcriptomes (**C**) while markers for neurons were all expressed at higher levels in the sympathetic neuroblast transcriptomes (**D**).

The transcriptomic profiles of sympathetic neuroblasts and adrenal chromaffin cells were determined by RNA sequencing in four biological replicates of FACS-isolated adrenal chromaffin precursors and sympathetic neuroblasts from E12.5 TH-Cre::R26R-EYFP mice. Sequencing yielded more than 30 million 50 base pair single-end informative reads per sample and 17,169 annotated genes were detected. Principal component analysis indicates the differences in transcriptomic profiles were mainly due to cell types, confirming a good segregation of adrenal chromaffin cells and sympathetic neuroblasts (Fig. 4A). When the differential transcriptome between adrenal chromaffin precursor cells and sympathetic neuroblasts was generated (Fig. 4B), 4,786 genes were differentially expressed with log2 fold change >1, and both false discovery rate (FDR) and *p*-value < 0.05. Fold change is calculated by the gene expression level in adrenal chromaffin cells divided by the gene expression level in sympathetic neuroblasts.

Droplet digital PCR (ddPCR) was used to confirm the RNA sequencing results. Thirteen genes that covered a range from high to low expression levels and fold changes were tested. For all of the genes, mRNA expression patterns by ddPCR were highly consistent with RNA sequencing results (Fig. S1) in that the fold changes of expression in adrenal chromaffin cells relative to sympathetic neuroblasts revealed by both methods were strongly correlated in linear regression analysis (r = 0.989).

The RNA sequencing data confirmed the cell type-specific gene expression of markers of mature sympathetic neurons and adrenal chromaffin cells (Fig. 4C,D). Messenger RNA for known adrenal chromaffin cell markers, expressed between 6 and 24-fold greater in chromaffin cells (Fig. 4C), included *Dbh* (dopamine beta hydroxylase)^18,19^, *Chga (*chromagranin A) and *Chgb (*chromagranin B)^20^ and *Vmat1* or *Slc18a* (vesicle monoamine transporter 1 or solute carrier family 18 member A1)^10,21,22^. Known markers of sympathetic neurons expressed at between 2.5 and 4-fold difference in sympathetic neuroblasts (Fig. 4D) included *Nefl (*neurofilament, light)^9,19,23^, *Nefm* (neurofilament, medium)^19,24^, *Vmat2* or *Slc18a2* (vesicle monoamine transporter 2 or solute carrier family 18 member A2)^10,25–27^, *Tuj1* or *Tubb3 (*Tubulin Beta 3 Class III),^20,28,29^, *Ret (*Receptor tyrosine kinase)^30,31^ and *Isl1* (Insulin gene enhancer protein)^10,22,27^. Of particular significance was *Cartpt* mRNA^17^, which was more than 8-fold enriched in sympathetic neuroblasts over adrenal chromaffin cells. E12.5 sympathetic neuroblasts have no detectable CART immunoreactivity (see Fig. 1I) so either the *Cartpt* mRNA is not translated into protein or any CART protein present is below the level detectable using immunohistochemistry. Expression of tyrosine hydroxylase (*Th*), a key enzyme required for catecholamine biosynthesis in both chromaffin cells and sympathetic neurons, was much (approximately 6-fold) higher in FACS-isolated adrenal chromaffin cells than sympathetic neuroblasts (Fig. 4C), consistent with TH protein immunohistochemistry.

### Gene Ontology and Pathway Analysis

Gene ontology (GO) analysis classified the transcriptome of E12.5 adrenal chromaffin cell and sympathetic neuroblasts into 17 and 28 GO functional terms respectively (Table 1). For the adrenal chromaffin cells, genes were abundant in biological processes that included; pattern specification processes, cell-cell signaling, system development, cell-cell adhesion and synaptic vesicle exocytosis. Among these, “pattern specification – dorsal/ventral axis specification” was the most highly over-represented term with 4.54-fold enrichment. For the transcriptome of sympathetic neuroblasts, biological processes such as chromatin organization, cell cycle, DNA metabolic process and regulation of gene expression – epigenetic were overrepresented. “Organelle organization – chromatin assembly” was the most enriched term with 8.46-fold. Most of these categories related to proliferation. This reflects the fact that sympathetic neuroblasts are highly proliferative at E12.5, in contrast to adrenal chromaffin cells^17^. We also employed the Ingenuity Pathway Analysis (IPA) to identify potential signaling pathways that are activated during chromaffin cells development (Fig. S2). In the top 15 representative pathways, most of the pathways are also related to proliferation and replication including; cell cycle control of chromosomal replication (top 1), cyclins and cell cycle regulation, mismatch repair in eukaryotes and cell cycle – G1/S checkpoint regulation. We also observed a group of cancer related pathways that included; molecular mechanisms of cancer, breast cancer regulation by Stathmin1, and ovarian cancer signaling. The G-Protein coupled receptor signalling is also overrepresented in the transcriptome.

**Table 1:** Gene ontology classification by overrepresentation test for enrichment analysis.

GO Term (GO ID)	Number of Observed Genes	Fold Enrichment	*p* Value
**High in chromaffin cells**			
**Developmental process (32502)**	**298**	**1.45**	**3.11e-08**
Pattern specification process (7389)	35	2.11	1.18e-02
Dorsal/ventral axis specification (9950)	12	4.54	5.08e-03
Cell communication (7154)	391	1.28	5.48e-05
Cell-cell signaling (7267)	91	1.9	2.96e-06
Transmembrane receptor protein tyrosine kinase signalling pathway (169)	46	2.33	6.36e-05
Synaptic transmission (7268)	59	1.81	3.92e-03
Intracellular signal transduction (35556)	148	1.43	3.64e-03
Signal transduction (7165)	335	1.21	4.10e-02
**Anatomical structure morphogenesis (9653)**	**36**	**2.15**	**6.74e-03**
**Ectoderm development (7398)**	**74**	**1.84**	**2.21e-04**
**System development (48731)**	**160**	**1.45**	**6.59e-04**
Nervous system development (7399)	107	1.58	1.18e-03
**Biological adhesion (22610)**	**99**	**2**	**4.76e-08**
Cell-cell adhesion (16337)	61	1.94	4.06e-04
**Others**			
Synaptic vesicle exocytosis (16079)	23	2.63	9.99e-03
Blood circulation (8015)	38	2.58	6.47e-05
**High in sympathetic neuroblasts**			
**Cellular component organization or biogenesis (71840)**	**229**	**1.9**	**3.21e-18**
Cellular component organization (16043)	213	1.92	2.96e-17
Organelle organization (6996)	141	2.7	5.18e-23
Chromatin assembly (31497)	15	8.46	1.79e-07
Chromatin organization (6325)	88	4.63	6.96e-29
Cytoskeleton organization (7010)	26	2.48	8.75e-03
Cellular component biogenesis (44085)	56	1.67	4.72e-02
**Cellular process (9987)**	**737**	**1.24**	**1.13e-11**
Chromosome segregation (7059)	39	5.15	1.04e-13
Cell cycle (7049)	145	2.45	6.48e-20
Mitosis (7067)	70	2.89	3.47e-12
Regulation of cell cycle (51726)	19	2.6	5.00e-02
**Metabolic process (8152)**	**620**	**1.31**	**4.71e-13**
Primary metabolic process (44238)	545	1.35	2.86e-13
Nucleobase-containing compound metabolic process (6139)	373	1.73	1.60e-24
DNA metabolic process (6259)	94	4.01	2.28e-26
DNA recombination (6310)	18	5.39	4.06e-06
DNA replication (6260)	43	4.32	1.41e-12
DNA repair (6281)	45	4.07	2.55e-12
RNA splicing, via transesterification reactions (375)	31	3.16	1.12e-05
mRNA processing (6397)	45	2.67	1.82e-06
mRNA splicing, via spliceosome (398)	38	3.01	1.24e-06
Regulation of nucleobase-containing compound metabolic process (19219)	61	1.63	4.71e-02
Nitrogen compound metabolic process (6807)	229	1.68	3.22e-12
Phosphate-containing compound metabolic process (6796)	124	1.45	7.03e-03
Biosynthetic process (9058)	147	1.42	4.25e-03
**Others**			
Regulation of gene expression, epigenetic (40029)	19	4.04	1.31e-04
Nuclear transport (51169)	21	3.11	1.93e-03

Gene ontology analysis was performed by PANTHER v.11^59^ statistical overrepresentation test with *Mus musculus* reference list. Transcriptomes for adrenal chromaffin cells and sympathetic neuroblasts with FC > 2 were tested separately. Only overexpressed term with *p* < 0.05 are shown.

### Genes that underlie adrenal chromaffin cell and sympathetic neuroblast development

In addition to the GO enrichment and pathway analysis that consider mainly the fold difference, our analysis also took into account of the transcript abundance for prioritizing genes of interest. For the 4,786 genes expressed at more than 2-fold difference across the two cell types and with FDR cutoff at 0.05, RNA sequencing provided a comparison of the relative numbers of copies present (as counts per million transcripts – CPM) and of the relative expression levels (fold difference) between adrenal chromaffin cells and sympathetic neuroblasts. Although it is difficult to predict from the number of copies alone whether a gene actively plays a role in development, genes that are strongly differentially expressed by one of the two cell types may be important genes in the development of that cell type. We therefore ranked genes by fold difference and in our initial analysis examined only genes with a CPM of 40 or more (33.55% of total genes detected). To confirm whether the expression of any gene selected was peaking at the adrenal chromaffin cell stage, rather than in bridge cells or in Schwann cell precursors, we examined the raw data (Accession No. GEO99933 at NCBI) by Furlan *et al*.^4^. Finally, attention was paid to transcription factors and genes forming signaling pathways as they are most likely to direct differentiation.

In adrenal chromaffin cells, of the 2,938 genes expressed at more than 2-fold difference and with FDR cutoff at 0.05, we identified five transcription factors (*Elf3*, *Elf4*, *Foxq1*, *Bhlhe40* and *Trim16*) and 13 genes likely to be involved in cell signaling (3 G-protein receptors; *Gipr*, *Casr* and *Gpr139*, 5 receptors; *Acvrl1*, *Baiap3*, *Dll4*, *Epha4* and *Procr*, a protein kinase; *Nrk*, a PI3k pathway member; *Dapp1*, a connexin; *Gjb5*, a secreted protein; *Wnt9a* and a regulator of G-protein signaling; *Rgs5*) (Table 2) as meeting these selection criteria. All of these genes were differentially highly expressed (130 to 9-fold) and, based on the raw data of Furlan *et al*.^4^ showed peak expression in developing adrenal chromaffin cells rather than bridge cells. None has been associated previously with chromaffin cell development^9,10^.

**Table 2:** Potential novel regulators of differentiation in chromaffin cells.

Gene symbol	Gene name	Fold Change	Average CPM		FDR
			Adrenal Chromaffin Cells	Neuroblasts	
*Elf3*	E74-like factor 3	129.61	70.21	0.53	2.91e-40
*Acvrl1*	Activin A receptor, type II-like 1	113.65	89.09	0.76	6.47e-43
*Gipr*	Gastric inhibitory polypeptide receptor	110.20	148.09	1.29	1.77e-56
*Casr*	Calcium-sensing receptor	77.26	79.92	0.98	1.88e-27
*Foxq1*	Forkhead box Q1	65.83	191.28	3.10	4.01e-58
*Nrk*	Nik related kinase	54.92	903.65	16.13	1.78e-11
*Baiap3*	BAI1-associated protein 3	53.49	426.34	7.87	1.14e-54
*Bhlhe40*	Basic helix-loop-helix family, member e40	52.96	220.64	4.21	3.50e-54
*Gjb5*	Gap junction protein, beta 5	50.57	102.94	2.23	9.09e-26
*Dapp1*	Adaptor for phosphotyrosine and 3-phosphoinositides	35.14	149.67	4.07	1.91e-33
*Epha4*	Eph receptor A4	33.56	61.58	1.73	2.20e-16
*Procr*	Protein C receptor, endothelial	31.54	81.68	2.46	3.03e-27
*Trim16*	Tripartite motif-containing 16	30.46	352.51	11.13	4.53e-56
*Gpr139*	G protein-coupled receptor 139	29.36	98.63	3.24	7.29e-31
*Rgs5*	Regulator of G-protein signalling	28.79	705.21	23.35	2.43e-28
*Wnt9a*	Wnt Family Member 9A	25.10	45.87	1.81	1.81e-24
*Dll4*	Delta-Like 4 (Drosophila)	21.08	40.86	1.61	3.19e-23
*Elf4*	E74-like factor 4	14.85	227.91	15.07	2.98e-23

Fold changes and mean expression were obtained using the Bioconductor edgeR package^58^ with variance between samples by trended estimate. Fold changes is calculated by the gene expression level in adrenal chromaffin precursor cells divided by the gene expression level in sympathetic neuroblasts. Mean expression is reported average count per million (CPM).

Among the 1,848 genes that were more highly expressed in sympathetic neuroblasts (Table 3) were five transcription factors (*Ebf1, Aff3*, *Hoxc8, E2f7 and Foxm1*), and 13 genes likely to be involved in cell signaling (1 receptor; *Islr2*, 3 signaling molecules; *Vip*, *Fgf13* and *Bmp3*, 5 genes involved in intracellular signaling; *Shcbp1*, *Crabbp1, Socs2, Lrr1* and *Fam83d* and 4 genes potentially involved in Rho GTPase signaling; *Arhgap22*, *Iqgap3*, *Depdc1b*, *Racgap1*). Many of the remaining genes highly expressed in sympathetic neuroblasts are involved in regulation of the cell cycle or the cytoskeleton.

**Table 3:** Potential regulators of differentiation in sympathetic neuroblasts.

Gene symbol	Gene name	Fold Change	Average CPM		FDR
			Adrenal Chromaffin Cells	Neuroblasts	
*Vip*	Vasoactive intestinal polypeptide	32.70	1.54	48.73	6.37e-32
*Islr2*	IgG superfamily containing leucine-rich repeat 2	13.21	4.55	58.97	3.40e-18
*Ebf1*	Early B cell factor 1	12.56	4.83	58.67	4.52e-12
*Crabp1*	Cellular retinoic acid binding protein I	8.97	76.93	674.68	7.13e-10
*Fgf13*	Fibroblast growth factor 13	8.22	12.64	100.82	2.68e-19
*Aff3*	AF4/FMR2 family, member 3	7.15	6.85	48.86	2.91e-11
*Hoxc8*	Homeobox C8	7.11	9.04	61.12	3.15e-08
*Lrr1*	Leucine rich repeat protein 1	6.62	6.87	43.23	6.23e-12
*Racgap1*	Rac GTPase-activating protein 1	6.38	68.13	420.41	3.65e-21
*Depdc1b*	DEP domain containing 1B	5.96	14.88	86.48	7.74e-15
*Socs2*	Suppressor of cytokine signaling 2	5.78	19.13	106.37	2.06e-15
*Iqgap3*	IQ motif containing GTPase activating protein 3	5.73	32.61	182.59	4.32e-16
*Fam83d*	Family with sequence similarity 83, member D	5.59	11.00	59.25	5.42e-11
*Shcbp1*	Shc SH2-domain binding protein 1	5.48	14.51	75.62	4.44e-01
*Arhgap22*	Rho GTPase activating protein 22	5.26	10.24	52.67	2.29e-09
*E2f7*	E2F transcription factor 7	5.11	10.60	52.48	3.47e-07
*Bmp3*	Bone morphogenetic protein 3	5.08	13.20	64.23	2.10e-08
*Foxm1*	Forkhead box M1	4.98	42.59	206.05	4.23e-14

Fold changes and mean expression were obtained using the Bioconductor edgeR package^58^ with variance between samples by trended estimate. Fold changes is calculated by the gene expression level in adrenal chromaffin precursor cells divided by the gene expression level in sympathetic neuroblasts. Mean expression is reported as average count per million (CPM).

### Potential Regulators are Transiently and Differentially Expressed During the Key Stage for Sympathoadrenal Development

**Figure 5:**
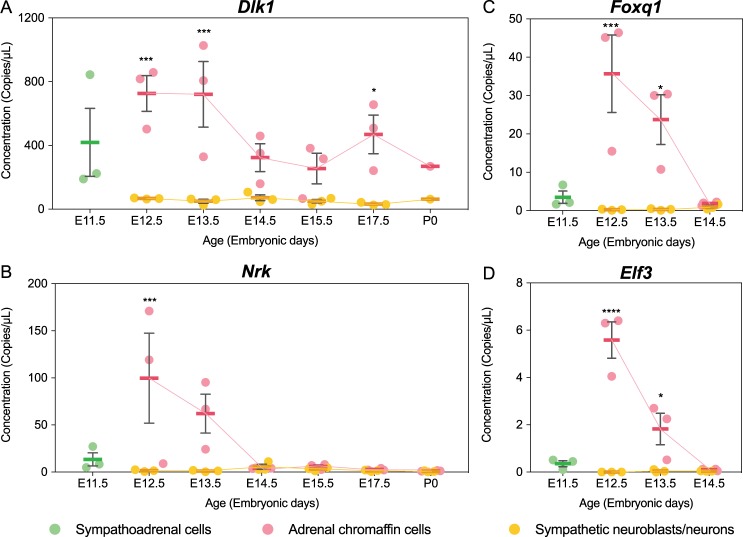
Temporal gene expression pattern of *Dlk1*, *Foxq*1, *Nrk* and *Elf3* for sympathetic neuroblasts/neurons (orange) and adrenal chromaffin precursors/cells (pink) after FACS based on differential expression of EYFP in THCre::R26REYFP embryos. (**A**,**B**) Gene expression pattern of *Dlk1* and *Nrk* were measured at E11.5 to P0. (**C**,**D**) Gene expression pattern of *Foxq1* and *Elf4* were measured at E11.5 to E14.5. Absolute concentration of mRNA copies per μL were measured and reported as individual measures (dot) and mean ± SEM (bar). In each case, 3 biological replicates were averaged, except on P0 for Dlk1 where only 1 biological replicate was measured. Asterisks indicate pairs of means that were significantly different using two-way ANOVA (*p < 0.05; **p < 0.01).

We then further investigated the temporal expression patterns of four genes by ddPCR gene expression analysis (Fig. 5). We chose *Dlk1*, which is expressed at very high levels (9,286 CPM) by developing chromaffin cells. The gene has been identified previously as being associated with developing chromaffin cells^9,10^ but it is not known whether it is developmentally regulated. From the novel genes in Table 2 we chose *Elf3* and *Foxq1* as the transcription factors with the highest fold difference, and *Nrk* as a novel and highly expressed kinase in the JNK pathway^32^. All of the genes were transiently expressed during chromaffin cell development with highest expression on E12.5 and declined thereafter, although *Dlk1* also had a second peak of expression on E17.5 before declining in expression for a second time. In every case, the expression of all of the genes was very low in sympathetic neuroblasts at all ages examined.

### *Nrk*-deficiency in Mice Impaired the Acquisition of an Adrenergic Phenotype by Chromaffin Cells and altered Cell Proliferation in Both Cell types

Among the potential regulators of chromaffin cell development, *Nrk* is expressed at the highest level in E12.5 chromaffin cells (Table 2), more than 54-fold higher than in sympathetic neuroblasts. To examine the function of *Nrk* in chromaffin cell development, *Nrk*-deficient mice^33^ were examined at E18.75, when development of the adrenal medulla and sympathetic ganglia are mostly complete. E18.75 *Nrk* mutants were generated by crossing heterozygous *Nrk* mutant female mice (*Nrk*-het) to *Nrk*-null mutant male mice. As a result, hemizygous male (*Nrk*^+/Y^) offspring are wild-type; heterozygous mutant females (*Nrk*^+/−^) contain approximately 50% of *Nrk*-null cells due to the random effect of X-inactivation in the wild-type *Nrk* allele; homozygous mutant females (*Nrk*^−/−^) and hemizygous mutant males (*Nrk*^−/Y^) are *Nrk*-null. Four embryos of each genotype across two litters were examined.

**Figure 6:**
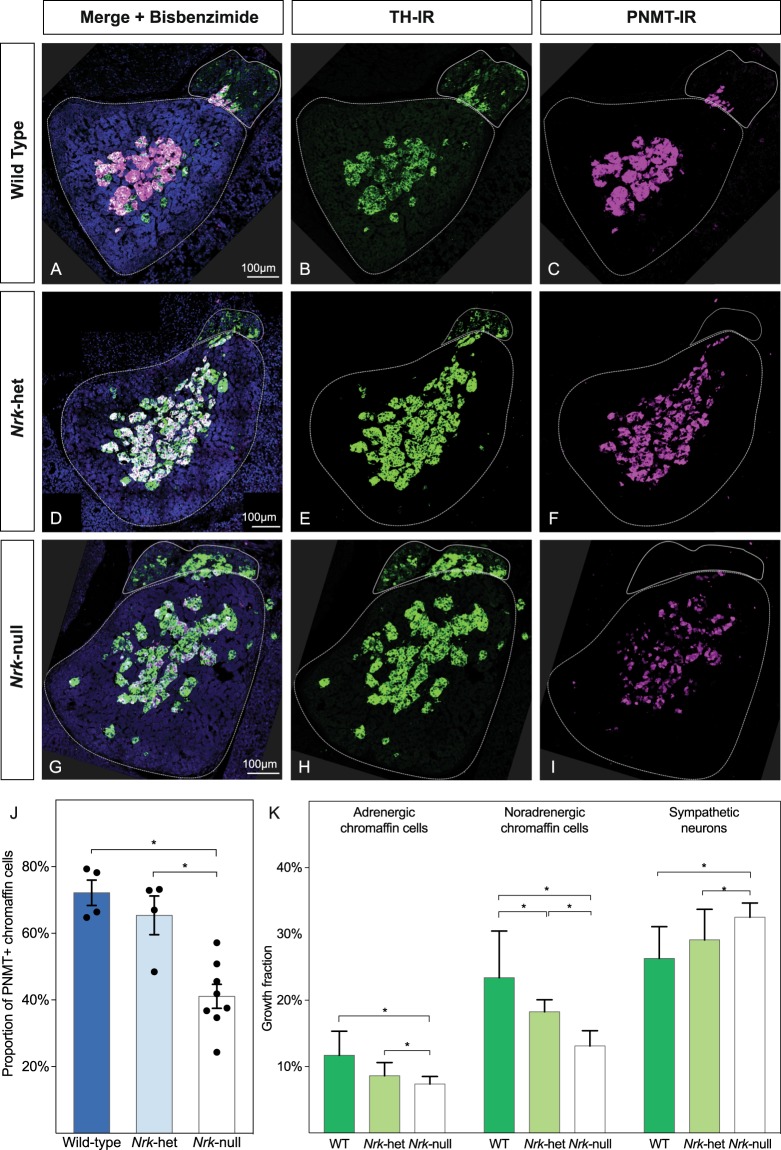
*Nrk* disruption in homozygous mutant led to a defect in adrenergic phenotype acquisition in the adrenal chromaffin cells. (**A**) Immunostaining of transverse sections through the adrenal region of E18.75 mouse embryos in wild type and *Nrk* mutant mice, showing TH (green) and PNMT (magenta) immunoreactivity. (**A**–**C**) Is from a wild type E18.75 mouse. (**D**–**F**) Show a section from an *Nrk*-het mutant mouse. (**G**–**I**) Is from an *Nrk*-null mutant mouse. The prevertebral suprarenal ganglia and the adrenal glands are outlined in a solid line and dashed line respectively, based on bisbenzimide (BB, cyan) staining (**A**,**D**,**G**). (**J**) Loss of *Nrk* reduces the proportion of adrenergic chromaffin cell in the adrenal glands. The number of TH+ noradrenergic adrenal chromaffin cells and their PNMT+ adrenergic subpopulation cells were counted in sections from wild-type, *Nrk*-het and *Nrk*-null (both *Nrk*^−/−^ female and *Nrk*^−/Y^ male) mutant mice with n = 4. The proportion of PNMT+ adrenergic chromaffin cells were calculated by number of PNMT+ (adrenergic) cells/number PNMT−, TH+ (noradrenergic) adrenal chromaffin cells and shown here as mean proportion ± SEM along with individual measures for each embryo. (**K**) Growth fractions for adrenergic chromaffin cells, noradrenergic chromaffin cells and sympathetic neurons were examined using Ki67 immunostaining for cycling cells in and calculated as proportion of total cells of each type.

In *Nrk* mutant mice, the size, appearance and anatomical position of the adrenal glands were not noticeably different from wild-type mice. The numbers of adrenal chromaffin cells and sympathetic neuroblasts (Fig. 6A–I) were examined in transverse sections of the upper abdomen at the adrenal level. Both cell types expressed TH, but only adrenal chromaffin cells were immunoreactive for phenylethanolamine N-methyltransferase (PNMT), the enzyme that generates adrenaline from noradrenaline. *Nrk*-null and *Nrk*-het mutant mice expressed TH in sympathetic neurons of the suprarenal ganglion and adrenal medullary chromaffin cells as in wild-type mice. Subpopulations of the TH-IR+ adrenal medullary chromaffin cells also co-expressed PNMT in both wild-type and *Nrk* mutant mice. The number of PNMT-IR+ cells as a proportion of TH-IR+ adrenal chromaffin cells was compared for each genotype (Fig. 6J). In wild-type mice, 72.2% of adrenal chromaffin cells were PNMT-IR+ (adrenergic), while in *Nrk*-null mice, only 41.1% of adrenal chromaffin cells was PNMT-IR+ (Fig. 6J). In *Nrk*-het mice, the proportion of adrenergic chromaffin cells was intermediate between the wild-type and *Nrk*-null with 65.4% of TH-IR+ cells in the adrenal medulla being PNMT-IR+. The apparent intensity of PNMT-IR in adrenal chromaffin cells varied in the same way, being highest in wild-type mice. The difference in proportions of PNMT-IR+ cells was significant (Chi-squared test, *X*^2^, 762, *p* = 0.05, df = 2, *N* = 10833, critical value = 5.99), confirming that the proportions of PNMT+ chromaffin cells among the wild-type, *Nrk*-het and *Nrk*-null mice are not equal. In order to test where any significant differences lay, the 2 × 3 contingency table was subdivided^34^ into 3, 2 × 2 tables (wild-type versus *Nrk*-null, wild-type versus *Nrk*-het and *Nrk*-het versus *Nrk*-null), which revealed that *Nrk*-null mice were significantly different from both wild-type and *Nrk*-het mice (Fig. 6J).

These results demonstrate that disruption of *Nrk* caused developmental defects in the acquisition of an adrenergic phenotype in adrenal chromaffin cells. To examine whether the reduction of adrenergic chromaffin cells was associated with a defect in cell proliferation, the proportion of adrenergic (TH+/PNMT+) chromaffin cells, noradrenergic (TH+/PNMT−) chromaffin cells and sympathetic neuroblasts that were cycling (Ki67 immunoreactive) was quantified. Analysis of the growth fraction (proportion of cycling to non-cycling cells) showed loss of *Nrk* caused significant changes in proliferative behavior in all cell types (Fig. 6K; Chi-squared test, adrenergic chromaffin cells *X*^2^, 20.6, *p* = 0.05, df = 2, *N* = 4680; noradrenergic chromaffin cells, *X*^2^, 58.96, *p* = 0.05, df = 2, *N* = 4212; sympathetic neuroblasts, *X*^2^, 17.27, *p* = 0.05, df = 2, *N* = 3103, in each case critical value = 5.99). As before, each 2 × 3 contingency table was broken down to 3, 2 × 2 tables and re-tested to determine which pairwise comparisons were significantly different. In all three cell types, *Nrk*-null were significantly different from wild-type and from *Nrk*-het. *Nrk*-hets were also significantly different from wild type animals for PNMT− chromaffin cells but not for PNMT+ chromaffin cells or from sympathetic neurons. Details of the statistical analyses are shown in Table S1.

Loss of *Nrk* in null mutant mice led to a significant (40–50%) decrease in growth fraction so that only about 7.4% and 13.1% of adrenergic and noradrenergic chromaffin cells respectively were dividing, compared to 11.7% and 23.4% respectively in wild-type mice (Fig. 6K). As the proliferation of both adrenergic and noradrenergic chromaffin cells changed to a similar extent, the reduction in the proportion of chromaffin cells with an adrenergic phenotype is unlikely to be due solely, or even mainly, to defects in proliferation.

In contrast to the situation in developing adrenal chromaffin cells, loss of *Nrk* in sympathetic neuroblasts resulted in a significantly increased growth faction from 26.3% to 32.5% (Fig. 6K). As was the case for the proportion of PNMT+ cells, *Nrk*-het mice showed effects on proliferation intermediate between wild-type mice and *Nrk*-null mice in all cell types (Fig. 6K). The changes in the proportion of proliferating cells for neuroblasts in *Nrk*-null mice occurred despite this cell type failing to express *Nrk*.

### Imprinted genes

The Imprinting Resource at Mousebook (http://www.mousebook.org/mousebook-catalogs/imprinting-resource) lists 151 imprinted genes in the mouse. Of these, our RNA sequencing survey detected 83, largely somatic genes with a few large non-coding RNA genes. Many of the genes not detected were microRNA or small nucleolar RNA, both of which would not have been retained by our RNA purification and detection methods. Of the 83 detected genes, 50 were up-regulated more than 2-fold in adrenal chromaffin cells. Only 9 genes were up-regulated more than 2-fold in sympathetic neuroblasts (Table 4). While more genes overall were upregulated in adrenal chromaffin cells than in sympathetic neuroblasts, imprinted genes represented 1.24% of all genes upregulated more than two fold in adrenal chromaffin cells but only 0.38% of genes upregulated more than two fold in sympathetic neuroblasts.

**Table 4:** Top 25 imprinted genes differentially expressed at least 2-fold in chromaffin cells over sympathetic neuroblasts in E12.5 mouse.

Gene symbol	Gene name	Fold Change	Average CPM		FDR
			Adrenal Chromaffin Cells	Neuroblasts	
*Dlk1*	Delta-like 1 homolog (Drosophila)	7.9	9286.8	1143.9	7.59e-13
*Th*	Tyrosine hydroxylase	8.2	5709.5	675.9	1.17e-11
*Cdkn1c*	Cyclin-dependent kinase inhibitor 1 C (P57)	7.9	2863.2	348.3	5.48e-15
*Ndn*	Necdin	2.3	2621.8	1126.5	4.77e-03
*Gnas*	Guanine nucleotide binding protein, alpha stimulating	5.2	2317.3	432.5	1.25e-13
*H19*	H19, imprinted maternally expressed transcript	26.8	844.0	30.1	2.50e-26
*Meg3*	Maternally expressed 3	6.5	833.0	119.8	3.97e-07
*Ddc*	Dopa decarboxylase	2.2	797.5	356.0	9.60e-04
*Igf2*	Insulin-like growth factor 2	27.6	692.6	23.9	2.35e-43
*Peg13*	Paternally expressed 13	2.3	622.7	268.3	3.58e-04
*Zdbf2*	Zinc finger, DBF-type containing 2	8.2	571.3	66.9	5.07e-06
*Peg3*	Paternally expressed 3	3.0	461.2	151.1	2.14e-02
*Ampd3*	Adenosine monophosphate deaminase 3	6.4	400.5	60.2	7.00e-17
*Kcnk9*	Potassium channel, subfamily K, member 9	5.6	350.3	61.7	6.23e-07
*Blcap*	Bladder cancer associated protein homolog	3.7	304.0	80.4	4.42e-09
*Peg10*	Paternally expressed 10	27.5	267.3	9.3	6.06e-20
*Magel2*	Melanoma antigen, family L, 2	5.4	206.8	37.3	1.20e-12
*Nap1l5*	Nucleosome assembly protein 1-like 5	3.5	177.3	48.3	6.29e-06
*Casd1*	CAS1 domain containing 1	2.9	163.5	53.7	5.88e-04
*Trappc9*	Trafficking protein particle complex 9	2.3	148.1	63.3	4.43e-04
*Sgce*	Sarcoglycan, epsilon	4.4	133.4	29.6	1.43e-12
*Asb4*	Ankyrin repeat and SOCS box-containing 4	24.4	119.9	4.7	2.17e-24
*Impact*	Impact, RWD domain protein	3.0	118.8	37.4	1.68e-04
*Rian*	RNA imprinted and accumulated in nucleus	2.8	115.3	39.1	1.56e-03
*Begain*	Brain-enriched guanylate kinase-associated	2.9	48.9	16.0	4.60e-05

## Discussion

In this study, we describe a technique for separating adrenal chromaffin and sympathetic neuron precursors in embryonic mice before they segregate anatomically, which allowed us to generate a resource of RNA sequencing data on genes differentially expressed by the two cell types, identify a role for *Nrk* in adrenal chromaffin cell development and produce evidence that many imprinted genes are upregulated during adrenal chromaffin cell development.

### Isolating live adrenal chromaffin cells and sympathetic neuroblasts from as early as E12.5

The separation of developing adrenal chromaffin cells from sympathetic neuroblasts in TH-Cre::R26R-EYFP mice by FACS depended on the differing intensity of the EYFP reporter in the two cell types. Consistent with our earlier study^17^, we showed that, compared to adrenal chromaffin cells, sympathetic neuroblasts show relatively low TH immunoreactivity; unexpectedly, we also showed that in TH-Cre::R26R-EYFP mice, sympathetic neuroblasts show relatively high intensity of the EYFP reporter compared to chromaffin cells. Why the inverse levels of TH immunostaining and EYFP intensity occur in the two cell types is unclear. It could be due to differences in the activity of the promoter in the endogenous TH gene versus the same promoter after recombination in the EYFP transgene. It is also possible that the handling of the foreign protein, EYFP, differs between the two cells or that the intrinsic cytoplasmic microenvironment of the two cell types differs sufficiently to alter the fluorescence of the EYFP reporter. Finally, the timing of the activation of the transgene may be different in the two cells and levels of EYFP may not yet be maximal in the cell type activating the gene last. It should be noted that the difference in intensity level of EYFP by the two cell types only lasts until E14.5, after which they are the same, which favors the latter explanation above. Whatever the explanation for the difference, it is sufficient to separate efficiently the two cell types at E12.5 and E13.5, so that the separated cells carry the correct molecular signature as previously determined by single cell PCR^4^. One advantage of isolating the cells using FACs based on expression of EYFP driven from the TH promoter is that this approach yields living embryonic cells that can cultured for further study.

### NRK gene

Our study showed that *Nrk* is transiently and highly expressed (903 CPM) in the developing mouse adrenal chromaffin cells on E12.5, but is expressed at only low levels (16.5 CPM) in sympathetic neuroblasts. *Nrk* is an X chromosome-linked gene that encodes for a Ser/Thr kinase^35^. The protein, NRK, also known as NESK, belongs to the Group I germinal center kinase (GCK) subfamily^32^. *Nrk* mRNA is expressed by the myotome during embryogenesis as well as in the spongiotrophoblast layer of the placenta, but is not expressed in any adult tissues with exception of the mammary gland of pregnant female mice^33,35,36^.

Like other group I GCKs, NRK appears to activate JNK signaling in model *in vitro* systems^32^. In this process it sits downstream of TNF receptor associated factor 2 (TRAF2) and upstream of MEKK1 and MKK4 and mediates the effect of TNF alpha^32^. Our data show that *Traf2*, *Mekk1* (as *Map3k1*) and *Mkk4* (as *Map2k4*) are all expressed at moderate levels in both adrenal chromaffin cells and sympathetic neuroblasts. The roles of NRK *in vivo* are poorly understood. A previous study in *Nrk* mutant mouse found that NRK is important for placental development and labor induction, while absence of NRK led to overgrowth of the spongiotrophoblast in the placenta^33^. It was suggested that the phenotype of spongiotrophoblast overgrowth was similar to that seen when another X-linked homeobox gene, *Esx1*, was knocked out, raising the possibility that NRK can act in the same pathway as *Esx1*^33^, but *Esx1* is not expressed in either adrenal chromaffin cells or sympathetic neuroblasts.

In our study, we found *Nrk*-deficient mice displayed a significant reduction in the proportion of adrenergic adrenal chromaffin cells in neonates. Thus, one possible action of NRK is to promote the differentiation of adrenergic adrenal chromaffin cells. On the limited evidence available, the differentiation and phenotype of noradrenergic chromaffin cells seemed unaffected by loss of NRK, except there were both less PNMT+ and more PNMT− chromaffin cells per section in the *Nrk*-null animals, which is consistent with the idea that PNMT− (noradrenergic) chromaffin cells were failing to adopt a PNMT+ (adrenergic) phenotype in the absence of *Nrk*. Furthermore, disruption of *Nrk* also lead to an increase in proliferation of sympathetic neurons but a decrease in proliferation of both adrenergic and noradrenergic chromaffin cells in E18.75 *Nrk*-null mice.

One puzzle is how loss of NRK exerts an action on sympathetic neuroblast proliferation. Levels of mRNA for *Nrk* were much higher in adrenal chromaffin cells (903 CPM) at E12.5 than in sympathetic neuroblasts (16 CPM) at the same age. Furthermore, expression of *Nrk* was only elevated in adrenal chromaffin cells at E12.5 and E13.5 and was at low levels in neuroblasts from E11.5 to P0. It is possible that the effect observed at E18.75 in adrenal chromaffin cells could have been exerted by the absence of *Nrk* in adrenal chromaffin cells at E12.5 and E13.5.

### Imprinted genes

In our study we showed that 50 imprinted genes were up-regulated more than 2-fold in adrenal chromaffin cells, but only 9 imprinted genes were up-regulated more than 2-fold in sympathetic neuroblasts. Imprinted genes have not previously been specifically associated with the developmental of neural crest-derived cells. Imprinted genes have been suggested to co-express as a gene network in a range of circumstances, including embryonic development, postnatal growth and stem cell regulation^37–39^, although there is no evidence of a dominant function(s) for imprinted genes^37^. Varrault *et al*.^38^ suggested that around 15 imprinted genes were commonly expressed together to regulate embryonic growth. Subsequent studies identified many of the same genes as also being involved in regulating postnatal growth and in the regulation of adult stem cells^39–41^. A largely similar, but not identical list of imprinted genes comprised the network in each of these studies^38–41^ and overlapping genes included *H19*, *Igf2*, *Ndn*, *Peg3*, *Cdkn1c*, *Mest, Dlk1*, *Gtl2* and *Grb10*. All are also highly differentially expressed in developing chromaffin cells in our data, suggesting that the same imprinted gene network is active. Recently, the putative imprinted gene network has been expanded to include a large proportion of known imprinted genes and it has been suggested that many non-imprinted somatic genes are also co-activated with the imprinted gene network^37^.

Among imprinted genes are a number of non-coding RNAs^42,43^ including the long non-coding RNA, *Meg3* (maternally expressed gene 3), and many microRNAs. Loss of *Meg3* and the microRNAs it controls leads to reduced neural differentiation in human embryonic stem cells^43^. Recently, microRNAs have been implicated in adrenal chromaffin cell fate determination^24^, as deletion of the RNAse, Dicer, which processes miRNAs, leads to a reversion of developing chromaffin cells to a more neuronal phenotype^24^.

**Figure 7:**
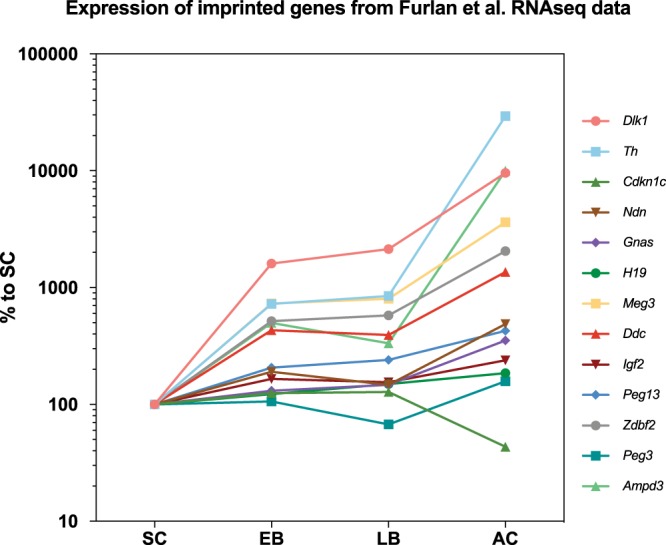
Data from Furlan *et al*.^4^, showing the expression of imprinted genes commonly expressed in differentiating cells. For each gene, values are normalised to expression levels in the Schwann cell precursor (SC). Note the logarithmic Y-axis. Nearly all genes are expressed at low levels in the Schwann cell precursor and in early (EB) and late (LB) bridge cells but at greatly increased levels in differentiating adrenal chromaffin cells.

Many of the same imprinted genes are also highly expressed in adrenal chromaffin cells in the raw data of Furlan *et al*.^4^. Analysis of the levels of imprinted gene expression across the four stages of adrenal chromaffin cell differentiation (Schwann cell precursor, early bridge cell, late bridge cell, adrenal chromaffin cell) in that data set suggests that, for most imprinted genes, expression is at very low or modest levels in Schwann cell precursors and at successively higher levels as differentiation proceeds (Fig. 7). A few imprinted genes were exceptions to this pattern; for instance, *Cdkn1c* was highest in Schwann cell precursors and decreased thereafter.

Overall, the importance of imprinted genes in development of adrenal chromaffin cells remains unclear. The silencing occurs by DNA-methylation and/or histone modification^44,45^ and is important for maintaining normal embryonic development and growth^46^. Deregulation of genomic imprinting gives rise to developmental disorders such as Beckwith-Wiedemann syndrome as well as tumorigenesis^47–49^. Most imprinted genes exist in chromosomal clusters called imprinted loci where the genes in each locus share an imprinting control region within the differentially methylated region^50,51^. The expression of imprinted genes may indicate that the development of adrenal chromaffin cells is a site of maternal vs paternal genome resource conflict, with different evolutionary pressures being exerted on the male and female genomes in this stage of development. Alternatively these genes are known to play developmental roles in other tissues and stages and imprinting may be the consequence of the role of these genes in the other tissues and limit proliferation. Expression of the network of imprinted genes often appears to correlate with withdrawal from the cell cycle and differentiation, perhaps to support subsequent tissue growth. More specific roles in embryonic development cannot be ruled out. One important differentially methylated region (DMR) includes the imprinted genes *Dlk1* and *Gtl2* (*Meg3*) and changes in expression or imprinting at this DMR is associated with a range of cancers, including neuroblastoma^52^. Changes in the expression of the imprinted microRNAs mir134a and mir335 have also been reported in many cases of neuroblastoma^5^. Given the upregulation of so many imprinted genes in developing adrenal chromaffin cells and the potential role of epigenetic dysregulation in neuroblastoma, imprinted genes represent novel targets for study.

## Materials and Methods

### Animals

All animal experiments were approved by the University of Melbourne Animal Experimentation Ethics committee and complied with National Health and Medical Research Council of Australia (NHMRC) guidelines. TH-IRES-Cre;ROSA26-EYFP (TH-Cre::R26R-EYFP) mouse embryos were generated by mating heterozygous TH-IRES-Cre mice with homozygous ROSA26-EYFP mice. The TH-IRES-Cre (nomenclature: B6.129X1-*Th*^*tm1(cre)Te*^/Kieg) mice were a kind gift from Prof A. Allen (University of Melbourne) that were originally obtained from Dr. T. Ebendal’s laboratory via the European Mutant Mouse Archive repository and maintained on a C57BL/6 background^53^. ROSA26-EYFP mice (nomenclature: B6.129X1-*Gt(ROSA)26Sor*^*tm1(EYFP)Cos*^/J) were obtained from Jackson Laboratories and were also maintained on a C57BL/6 background. *Nrk* mutant mice (nomenclature: B6.129S4-*Nrk*^*tm1Mkom*^) were generated by crossing *Nrk-*null males with *Nrk*-het females and maintained on a C57BL/6 background. Wild-type (WT) C57BL/6 mice were used for validation experiments. All mice were time plug-mated and the morning of detection of the plug was counted as embryonic day (E) 0.5. Pregnant dams at 11.5–14.5 days post-fertilization were killed by cervical dislocation and the embryos were collected and decapitated. See Supplementary data for genotyping and phenotyping methods.

### Tissue Dissection

Embryos were dissected in ice-cold “dissecting medium” (DMEM/F-12, Gibco, cat. 11320-033 supplemented with 19 m*M* HEPES, Sigma-Aldrich, cat. H0887. For E11.5 and E12.5 embryos, the viscera adjacent to the urogenital tract including the liver, intestines and stomach were first removed. The urogenital ridges and the tissues in between, including the dorsal aorta were then detached from the dorsal body wall by inserting the tips of fine forceps underneath, and pulled away by holding the dorsal aorta. In E11.5 tissues, the urogenital ridges were removed from the tissue of interest. In E12.5, the tissue of interested was further sub-dissected by removing the urogenital ridges, the kidneys and the paravertebral sympathetic chains, if attached. For E13.5 and E14.5 embryos, the urogenital tract was dissected according to Buehler *et al*.^54^, with modifications. Briefly, the viscera from the liver down to the genital tubercle were pulled away from the dorsal body wall by inserting fine forceps underneath the dorsal aorta at the level of the heart. In E13.5 tissues, this tissue mass was further microdissected to retain just the region containing the adrenal anlagen and the adjacent tissue surrounding the dorsal aorta. By E14.5, the adrenal glands and sympathetic ganglia were present with clearer anatomical segregation. Therefore, in E14.5–P0 tissues, the adrenal glands and the surrounding sympathetic ganglia were separately sub-dissected into different samples. Comparable tissues were also dissected from EYFP negative (−) embryos for compensation controls in the FACS. Tissues from 5–7 embryos were cut into smaller pieces and pooled into a 15 mL centrifuge tube (Corning) with 4 mL of ice-cold dissecting medium.

### Cell Dissociation and FACS Isolation

The tissues were then dissociated in 200 *µ*L embryo^−1^ Accumax (Innovative Cell Technologies) followed by incubation in a 37 °C incubator for 45 min. 1 mL of quench medium (dissecting medium supplemented with 10% FBS, Life Technologies; 100 units mL^−1^ penicillin and 100 *µ*g mL^−1^ streptomycin, Gibco; 2 m*M* Glutamax, Thermo Fisher; 37.5 *µ*g mL^−1^ DNase, Sigma-Aldrich) was added to each tube to stop the enzymatic activity and the tissues were triturated gently until completely dissociated. The dissociated cells were filtered through a 70 *µ*m nylon Cell Strainer (Falcon). The tube and filter was then washed with 1 mL of quench 1:5 medium (quench medium supplemented with 7.5 *µ*g mL^−1^ DNase) to release any remaining cells and the cells in the filtrate were pelleted by centrifugation at 250 g, 4 °C for 5 min. The pellet was re-suspended in 500 *µ*L quench 1:5 medium and viability dye, 7-Aminoactinomycin D (7-AAD, BD Pharmingen) was added at 1:1000 (v/v) dilutions to label dead cell for FACS. Compensation controls were also prepared with 200 *µ*L EYFP− cell suspension (double negative), 200 *µ*L EYFP− cell suspension labelled with 0.2 µL 7-AAD (7-AAD only) and 200 *µ*L 1:50 (v/v) dilution EYFP+ cell suspension (EYFP only).

Cells were filtered through a 35 *µ*m cell strainer cap again into a 5 mL polystyrene round bottom tube (Falcon) prior to FACS. Flow cytometry analysis and cell sorting were performed on a BD Influx or BD FACSAria Fusion cell sorter with a 100 *µ*m nozzle at 20 psi (BD Biosciences). The compensation controls were evaluated to optimize the voltages and gating for sorting. Live EYFP+ single cells were first isolated from dead cells on the basis of 7-AAD (excitation: 488 nm, emission 692/40 nm) and EYFP (excitation: 488 nm, emission 530/40 nm) intensity. The populations from E12.3 and E13.5 samples were then further analysed on the basis of side-scattered light (SSC) and EYFP intensity and sorted into EYFP+_Lo_ and EYFP+_Hi_ populations with the elimination of cells lying in between. For the live EYFP+ cells from E11.5 and the separated adrenal gland and sympathetic ganglia samples in E14.5–P0, a single homogenous population was observed so that no further FACS gating was needed. Dot-plots were generated by BD FACS^TM^ Sortware software. The cells were sorted directly into 750 *µ*L ice-cold TRIzol® LS reagent (Ambion) in a 1.5 mL nuclease-free LoBind microcentrifuge tube (Eppendorf) for RNA extraction.

### RNA Extraction and Droplet Digital PCR

Total RNA from isolated EYFP+_Hi_ and EYFP+_Lo_ cells were extracted using by TRIzol/chloroform extraction followed by RNeasy Micro Kit (Qiagen). RNA pellets with ~20,000 cells from more than 10 embryos with a sex ratio within a range of 1:1.3 (male:female or female:male) were pooled. Total RNA quality and quantity from each sample were analysed on a Bioanalyzer 2100 (Agilent) and the RIN numbers of all samples were >8 with average RIN = 9.7. For Droplet digital PCR, cDNA was synthesized using the iScript Advanced cDNA Synthesis Kit (Bio-Rad Technologies). Droplet digital PCR was performed in biological triplicates and technical duplicates on a QX100 Droplet Digital PCR system (Bio-Rad Technologies) with TaqMan Assays primer/probe mixture (Thermo Fisher Scientific). See Supplementary data for detail method.

### RNA Sequencing

Four biological replicates of each EYFP+_Hi_ and EYFP+_Lo_ paired RNA sample were generated for transcriptomic analysis. cDNA library was prepared by random priming using the SMART-seq v4 Ultra Low Input RNA Kit (Clontech, Cat. 634889) followed by PCR amplification with the addition of Illumina adaptors for multiplexing experiment using Nextera XT Kit (Illumina, Cat. FC-131-1096). Sequencing was performed with 50 base pair single-end reads on an Illumina HiSeq 2500 platform.

### Bioinformatics Analysis

Samples from RNA-seq were demultiplexed and raw reads were generated in fastq format by HiSeq Analysis Viewer software (Illumina). Around 29 million reads were detected per sample. Quality assurance and quality control for all samples were evaluated using FastQC analysis. The effect size of biological versus technical variances were analyzed by principal component analysis. Reads were aligned with HISAT2^55^ to the Genome Reference Consortium mouse genome, GRCm38 with gencode consortium gene annotation M8^56^. Reads were assigned to genes using HTSeq^57^. Differential expression between adrenal chromaffin precursor cells and sympathetic neuroblasts was obtained using the Bioconductor edgeR package^58^. Fold changes were calculated by the gene expression level in adrenal chromaffin cells divided by the gene expression level in sympathetic neuroblasts. Genes with fold change greater than 2 and *p*-value of less than 0.05 after Benjamini-Hochberg false discovery rate correction were considered significantly differentially expressed. Gene ontology analysis for genes higher in chromaffin precursor cells and sympathetic neuroblasts of the differential transcriptomes was performed by PANTHER v.11^59^ statistical overrepresentation test with *Mus musculus* reference list. Ingenuity Pathways Analysis v.01–06 tool was employed to the differential expressed genes in chromaffin cells to sympathetic neuroblasts for gene set and signaling pathways enrichment analysis.

### Immunohistochemistry and Data Analysis

Immunohistochemistry followed the methods described in^17^. See Supplementary data for antisera used. For comparison of TH, CART and EYFP immunoreactivity, confocal images were taken on a Zeiss Meta 501 scanning confocal microscope. For comparing TH, Ki67 and PNMT immunoreactivity in *Nrk* mutant mice, confocal images were taken on a Zeiss LSM 800 confocal microscope. All images were processed by Zeiss Image Browser (v4.0.0241, Carl Zeiss Microimaging). Graphs were prepared by using Numbers software (version 3.6.2, Apple Inc.) or Prism software (version 7.0a, GraphPad). Statistically analysis was performed with Prism 7 software using two-way ANOVA for gene expression pattern, linear regression for correlation analysis, and chi-squared test for *Nrk* loss-of function analysis.

## Supplementary information

Supplementary information

## Data Availability

RNA-seq data are available in the ArrayExpress database at EMBL-EBI (www.ebi.ac.uk/arrayexpress) under accession number E-MTAB-7086.
