# Supplementary material for: RNA-seq of Isolated Chromaffin Cells Highlights the Role of Sex-Linked and Imprinted Genes in Adrenal Medulla Development
Source: Sci Rep. 2019 Mar 8;9:3929. doi: 10.1038/s41598-019-40501-0 (PMC6408553; doi:10.1038/s41598-019-40501-0)
Supplement: Supplementary file 1 — Supplementary information [file 41598_2019_40501_MOESM1_ESM.docx]

**RNA-seq of Isolated Chromaffin Cells Highlights the Role of Sex-Linked and Imprinted Genes in Adrenal Medulla Development**

Wing Hei Chan^1^*, Masayuki Komada^2^, Toshiaki Fukushima^2^, E. Michelle Southard-Smith^3^, Colin R Anderson^1^, Matthew J Wakefield^4, 5^*

^1^Department of Anatomy and Neuroscience, University of Melbourne, Australia, ^2^Cell Biology Center, Institute of Innovative Research, Tokyo Institute of Technology, Japan, ^3^Department of Medicine, Vanderbilt University School of Medicine, Nashville, Tennessee, ^4^Melbourne Bioinformatics, University of Melbourne, Australia, ^5^Walter and Eliza Hall Institute, Australia

Supplementary data

Materials and Methods

Genotyping and Phenotyping

For genotyping, polymerase chain reaction (PCR) was performed using genomic DNA extracted from mouse tails. Heterozygous TH-IRES-Cre mice were genotyped with primers: *Cre*-Fw 5’-TGC AAC GAG TGA TGA GGT TC-3’ and *Cre*-Rv 5’-GCT AAC CAG CGT TTT CGT TC-3’ (GeneWorks). Genotyping for *Nrk* mutant mouse embryos, was performed using primers: *Nrk*-Fw 5’-CTC TCC CGA ACC ACA AAC CC-3’ and *Nrk*-Rv 5’-CAG GTA CCC ATG CGC TAG AC-3’. The sex of each EYFP+ embryo was determined using the method described by Lambert et al. ^61^ with primers: SRY-8276 5’-TGG GAC TGG TGA CAA TTG TC-3’; SRY*-*8677 5’-GAG TAC AGG TGT GCA GCT CT-3’ (GeneWorks) for the Y-chromosome. The phenotype of each TH-Cre::R26R-EYFP embryo was confirmed by the presence of EYFP positive (+) neurons in a squash preparation of the brain stem viewed under a Zeiss M1 fluorescent microscope.

RNA Extraction and Quantification

All RNA handling procedures were performed using RNAseZap (Ambion) treated equipment and nuclease-free barrier tips (Axygen) and RNase-free low-binding tubes (Eppendorf). Total RNA from isolated EYFP+_Hi_ and EYFP+_Lo_ cells were first extracted by TRIzol/chloroform extraction. Briefly, the cell lysate in TRIzol® LS was brought up to 1 mL with nuclease-free water (Ambion) followed by adding 75 *µ*L of RNase-free glycogen (Ambion). 200 *µ*L of chloroform was then added and vortexed thoroughly. Phase separation was performed in a Phase Lock Gel tube (5 PRIME) to minimize genomic DNA contamination. Total RNA in the upper aqueous phase was precipitated with 550 *µ*L ice-cold isopropanol at 12,200 g, 4ºC for 15 min. The RNA pellets were washed with 1 mL 80% ethanol and stored at –80ºC before further sample pooling and clean up. RNA pellets with ~20,000 cells from more than 10 embryos with a sex ratio within a range of 1:1.3 (male:female or female:male) were pooled and re-dissolved in 30 *µ*L nuclease-free water (Ambion). 2 *µ*L of DNase1 and SUPERase-In (Ambion) were added into the total RNA sample with 3.7 *µ*L DNase 10X buffer (Ambion) followed by a 30 min incubation at 37ºC for DNase digestion. The reaction was stopped by adding 100 *µ*L RLT/BME (1 *µ*L 2-mercaptoethanol, Sigma-Aldrich; 100 *µ*L RLT buffer, Qiagen) followed by adding 100 *µ*L of 70% ethanol. The total RNA sample was purified using RNeasy Micro Kit (Qiagen) according to the manufacturer’s instructions. Purified total RNA was eluted in 30 *µ*L RNase-free water (Qiagen). Total RNA quality and quantity from each sample were analysed on a Bioanalyzer 2100 (Agilent) and the RIN numbers of all samples were > 8 with average RIN = 9.7.

Complementary DNA Preparation and Droplet Digital PCR

cDNA was synthesized from 4.2 ng of total RNA using the iScript Advanced cDNA Synthesis Kit (Bio-Rad Technologies) according to the manufacturer’s instructions. Reverse transcription was performed at 42ºC for 30 min followed by 5 min inactivation at 85ºC. 1 *µ*L of cDNA sample was mixed with 24 *µ*L Supermix solution containing 12.5 *µ*L ddPCR Supermix for Probes (Bio-Rad Technologies) and 1.25 *µ*L TaqMan Assays primer/probe mixture (Thermo Fisher Scientific) followed by droplet generation. Droplet digital PCR (ddPCR) was performed in biological triplicates and technical duplicates on a QX100 Droplet Digital PCR system (Bio-Rad Technologies) with thermal cycle as follows: 95ºC for 10 min, 40 cycles of 94ºC for 30 second followed by 60ºC for 1 min, and 98 ºC for 10 min on a C1000 Touch thermal cycler (Bio-Rad Technologies). Results were analyzed using the Bio-Rad QuantaSoft v1.2 software with thresholds manually set. TaqMan Assays primer/probes used were: *Bmpr1b* (Mm03023971_m1), *Cartpt* (Mm04336402_m1), *Dll4* (Mm00444619_m1), *Dlk1* (Mm00494477_m1), *Dlx1* (Mm00438424_m1), *Dlx2* (Mm00438427_m1), *Elf3* (Mm01295975_m1), *Elf4* (Mm01321797_m1), *Foxq1* (Mm01157333_s1), *Fzd10* (Mn00558396_s1), *Gapdh* (Mm99999915_g1), *Msx2* (Mm00442992_m1) and *Nrk* (Mm00479081_m1).

Immunohistochemistry

TH-Cre::R26R-EYFP embryos in E12.5 and E13.5 were fixed overnight in Zamboni’s fixative (2% formaldehyde and 15% saturated picric acid in 0.1 *M* phosphate buffer, pH 7.4, composition in m*M*; Na_2_PO_4_, 75; NaH_2_PO_4_·2H_2_O, 25). For *Nrk* mutant and hemizygote wildtype, E18.75 mice were fixed overnight in 4% formaldehyde. Fixed embryos were stored in 20% sucrose solution at 4ºC. Abdominal region of embryos containing the adrenal glands were embedded in O.C.T. (TissueTek) and then snap frozen in liquid nitrogen-cooled isopentane. Transverse sections were cut on a cryostat at 10 *µ*m thickness and processed for immunostaining by antisera (Table S2).

Sections of TH-Cre::R26R-EYFP embryos were processed overnight with primary antisera in a combination of rabbit anti-CART (1:500, Phoenix Pharmaceuticals), chicken anti-TH (1:100, Millipore), and Goat anti-GFP (1:400, R&D Systems). After washing in phosphate buffered saline (PBS, composition in m*M*; NaCl, 145; Na_2_PO_4_, 7.5; NaH_2_PO_4_·2H_2_O, 2.5) the bound primary antisera were visualized after two hours incubation with secondary antisera: donkey anti-rabbit biotin (1:400, Jackson), donkey anti-chicken DyLight 594 (1:1000, Jackson ImmunoResearch), and donkey anti-sheep Alexa Fluor 647 (1:500, Invitrogen) and a 1 hour tertiary step with streptavidin conjugated to DyLight 405 (1:100, Jackson ImmunoResearch).

Sections for E18.75 *Nrk* mutant and wildtype mice were subjected to antigen retrieval by heating at 95ºC for 10 min in 0.01 *M* citrate buffer, pH 6.0 prior to primary antisera incubation and then exposed to chicken anti-TH (1:100, Millipore), rabbit anti-Ki67 (1:200, Thermo Scientific) and sheep anti-PNMT (1:500, Howe). After washing in PBS, the bound primary antisera were visualized after two hours incubation with secondary antisera: donkey anti-chicken FITC (1:100, Jackson ImmunoResearch), donkey anti-rabbit DyLight 594 (1:500, Jackson ImmunoResearch) and donkey anti-sheep Alexa Fluor 647 (1:500, Invitrogen), and the nuclei were stained with bisbenzimide (1 *µ*g/mL, Hoechst 33446).

Supplementary Figures and Tables


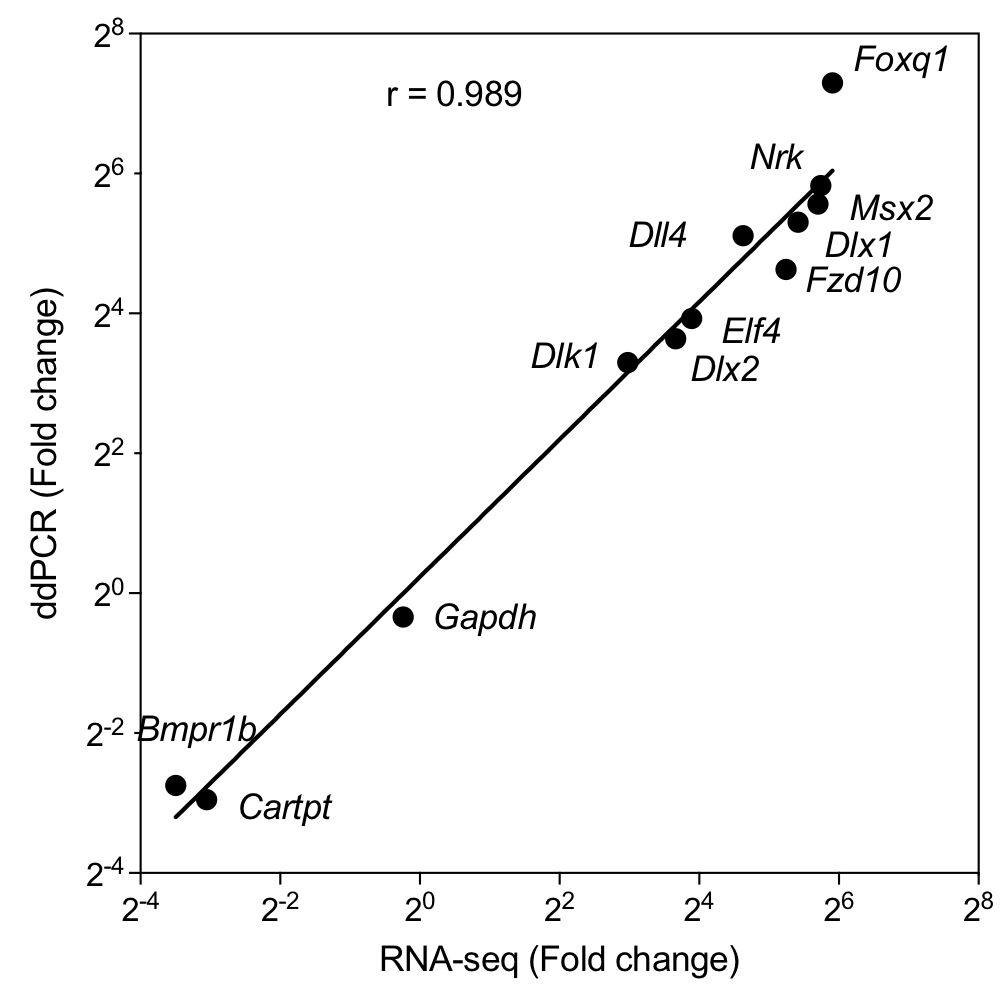


***Figure S1. Validation of RNA-seq by ddPCR for 11 of the 12 selected genes.*** *The RNA-seq data were verified by ddPCR with 11 selected genes covering high and low expression levels and fold changes plus the widely-used house keeper gene Gapdh. Note there were no copies of transcript for Elf3 detected in the sympathetic neuroblasts by ddPCR in agreement with RNA-seq, therefore its fold change between cell types could not be calculated and reported. The fold change of expression in adrenal chromaffin cells to sympathetic neuroblasts revealed by RNA-seq strongly correlated with ddPCR result (r = 0.978, p < 0.01).*

***Figure S2. Top 15 representative pathways identified by Ingenuity Pathway Analysis (IPA). RNA-seq revealed*** *DEGs of adrenal chromaffin cells compared to neuroblasts were analysed by IPA Core Analysis that top 15 significant overrepresented pathways are shown with minimum significance level (Threshold) at 1.25 –log(p-value). The ratio of mapped DEGs over the total number of molecules within the canonical pathway are shown with line plot (Orange).*

**Table S1. Pairwise Chi-squared analysis of proliferation in E18.75 PNMT− and PNMT+ chromaffin cells and in sympathetic neurons.**

**Table S2. List of antisera and fluorescence dyes used.**

| Antisera/Dye | Host | Dilution | Supplier | Cat/Lot number |
| --- | --- | --- | --- | --- |
| Primary |  |  |  |  |
| CART | Rabbit | 1:200 | Phoenix | H-003-62/01251-6 |
| GFP | Goat | 1:400 | R&D Systems | − |
| Ki67 | Rabbit | 1:200 | Thermo Fisher | − |
| PNMT | Sheep | 1:500 | P. Howe | − |
| TH | Chicken | 1:100 | Millipore | AB9702/2049252 |
| Secondary |  |  |  |  |
| Chicken DyLight 594 | Donkey | 1:1000 | Jackson | 703-516-155/- |
| Chicken FITC | Donkey | 1:100 | Jackson | 703-095-155/88953 |
| Rabbit Biotin | Donkey | 1:400 | Jackson | 711-065-152/95129 |
| Rabbit DyLight 594 | Donkey | 1:500 | Jackson | 711-515-152/99212 |
| Sheep Alexa Fluor 647 | Donkey | 1:500 | Invitrogen | A21448/404236 |
| Non-antisera Dye |  |  |  |  |
| Bisbenzimide (Hoechst 33442) | − | 100 ng mL^-1^ | Sigma | B2261 |
| Streptavidin DyLight 405 | − | 1:100 | Jackson | 016-470-084/98327 |
